# Simonkolleite Coating on Poly(Amino Acids) to Improve Osteogenesis and Suppress Osteoclast Formation in Vitro

**DOI:** 10.3390/polym11091505

**Published:** 2019-09-16

**Authors:** Shuyang Li, Xingtao Chen, Xiaomei Wang, Yi Xiong, Yonggang Yan, Zhi Tan, Xiaoyu Yang, Yuanye Li

**Affiliations:** 1 College of Physics, Sichuan University, Chengdu 610065, China; lisy370@hotmail.com (S.L.); chenxingtao007@126.com (X.C.); yixiong818@outlook.com (Y.X.); 2 Collaborative Innovation Center of Tissue Repair Material of Sichuan Province, College of Life Sciences, China West Normal University, Nanchong 637009, China; xiaomeiwang70@163.com; 3 Chengdu Customs Technology Center, Chengdu 610041, China; mitotan@163.com; 4 College of Veterinary Medicine, Sichuan Agricultural University, Chengdu 611130, China; abtcyxy@126.com (X.Y.); LyyLmFjy12@163.com (Y.L.)

**Keywords:** simonkolleite, coating, osteogenesis, osteoclast differentiation, bacteriostasis

## Abstract

Zinc can enhance osteoblastic bone formation and stimulate osteogenic differentiation, suppress the differentiation of osteoclast precursor cells into osteoclasts, and inhibit pathogenic bacterial growth in a dose-dependent manner. In this study, simonkolleite, as a novel zinc resource, was coated on poly (amino acids) (PAA) via suspending PAA powder in different concentrations of zinc chloride (ZnCl_2_) solution, and the simonkolleite-coated PAA (Zn–PAA) was characterized by SEM, XRD, FT-IR and XPS. Zinc ions were continuously released from the coating, and the release behavior was dependent on both the concentration of the ZnCl_2_ immersing solution and the type of soak solutions (SBF, PBS and DMEM). The Zn–PAA was cultured with mouse bone marrow stem cells (BMSCs) through Transwell^TM^ plates, and the results indicated that the relative cell viability, alkaline phosphatase (ALP) activity and mineralization of BMSCs were significantly higher with Zn–PAA as compared to PAA. Moreover, the Zn–PAA was cultured with RAW264.7 cells, and the results suggested an inhibiting effect of Zn–PAA on the cell differentiation into osteoclasts. In addition, Zn–PAA exhibited an antibacterial activity against both *S. aureus* and *E. coli*. These findings suggest that simonkolleite coating with certain contents could promote osteogenesis, suppress osteoclast formation and inhibit bacteria, indicating a novel way of enhancing the functionality of synthetic bone graft material and identifying the underline principles for designing zinc-containing bone grafts.

## 1. Introduction

Zinc, an essential trace element supporting the activity of numerous enzymes and participating in the bone metabolism [1,2], is well accepted as possessing the abilities to promote bone formation via stimulating osteoblastic differentiation, leading to an enhanced extracellular matrix (ECM) mineralization [3]. Many studies suggested that zinc can improve osteogenic differentiation by up-regulating the expression of various genes such as alkaline phosphatase (ALP), collagen type I (Col-I), osteocalcin (OCN) and osteopontin (OPN), thus improving the collagen synthesis and mineral deposition for bone regeneration [4,5]. Furthermore, zinc has also been reported to have an effect, not only in the resorption activity of osteoclasts but also in the formation of osteoclasts from macrophages [6,7]. The behavior of zinc toward the skeletal system is highly dose-dependent [8]. Zinc deficiency is thought to have positive correlation with osteoporosis and retarded skeletal development [9]. On the other hand, a high concentration of zinc can decrease ECM mineralization due to the inhibition of calcium deposition, and it can cause cytotoxicity [10,11]. For these reasons, the incorporation of zinc into a bone implant can be beneficial to bone regeneration under the condition that the release behavior and concentration of zinc is appropriate.

Simonkolleite (Zn_5_(OH)_8_Cl_2_·H_2_O) [12], also named tetrabasic zinc chloride or zinc chloride hydroxide, is a biocompatible mineral built with a structure involving stacked two-dimensional layers [13]. It has been used as a nutritional feed additive to supplement zinc for livestock due to a high bioavailability and antimicrobial activity [14]. Simonkolleite is also found to deposit on the surface of implanted metallic zinc, but little research has focused on its zinc release behavior and the resulting biological response [15,16]. As a zinc-containing mineral, simonkolleite may enhance bone regeneration due to the sustained release of zinc into the osseous microenvironment [17]; thus, simonkolleite coating on bone implants or fillings can be particularly helpful. In addition, as reported, simonkolleite can be precipitated from zinc chloride solution [18,19,20], so that immersing the substrate materials with basic groups into a zinc chloride solution to allow the simonkolleite to precipitate on a solid surface might be a simple and effective way to prepare simonkolleite coating. Poly(amino acids) (PAA), fabricated by an in-situ melting method, are currently used as an organic phase in composites for bone reconstruction [21], exhibiting not only suitable mechanical properties but also tailorable degradation rates for bone regeneration [22]. Though it has excellent properties in terms of being a non-toxic, low-cost, simple synthetic process with easy processing [23], it is biologically inert, which may induce fibrous encapsulation, hindering the osteogenesis process [24]. Therefore, we hypothesized that simonkolleite coating on PAA may endow PAA with the abilities to stimulate osteogenic differentiation and mineralization, and may suppress osteoclastic bone absorption, which may not only avoid fibrous encapsulation but more importantly may accelerate osteogenesis.

In this study, simonkolleite, as a novel zinc resource for bone regeneration, was coated on PAA via suspending PAA powders in different concentrations of a zinc chloride (ZnCl_2_) solution. X-ray diffraction (XRD), Fourier transform infrared spectroscopy (FT-IR) and X-ray photoelectron spectroscopy (XPS) were employed to characterize the coating. The aim of this research was to investigate the influence of simonkolleite coating on osteogenesis and osteoclastogenesis in vitro and on the antibacterial activity, and to further identify the underlying principles in order to design zinc-containing coatings on bone grafts.

## 2. Materials and Methods

### 2.1. Preparation and Characterization of Zn–PAA

The PAA was synthesized by the melt polymerization method. Briefly, 91.82 g *ε*-aminocaproic acid (0.70 mol, Xi’an Haixin Pharmaceutical and Chemical Co., Ltd., Xi’an, China), 24.00 g *γ*-aminobutyric acid (0.23 mol, Liaoning Kaiyuan Hengtai Chemical Co., Ltd., Shenyang, China), 5.00 g *L*-proline (0.04 mol, Hebei Kairuijie Amino Co., Ltd., Xintai, China), 3.00 g *L*-lysine (0.02 mol, Hebei Kairuijie Amino Co., Ltd., Xintai, China), 1.47 g *l*-glutamic acid (0.01 mol, Chengdu Kelong Co., Ltd., Chengdu, China), 50.0 mL water and 0.5 mL (50 vol. %) phosphorous acid (Sigma–Aldrich Co., LLC., St. Louis, MO, USA) were added in a three-necked flask with continuous stirring at 190 °C for 1 h and 210 °C for 2.5 h, sequentially. After cooling at room temperature, the PAA was smashed (YJ–500A, Jinan, China) into powder and sieved through a 100-mesh sieve for further experiments. The intrinsic viscosity (*η*) of the obtained PAA was 116.1 dL/g. The sieved PAA powder was dispersed into zinc chloride solutions with different concentrations (0.1, 0.05 and 0.025 mol/L, abbreviated as PAA–0.1M, PAA–0.05M and PAA–0.025M, respectively) at a mass-volume ratio of 20 g/L with continuous stirring for 20 h to prepare the simonkolleite layer coating. After being washed five times with deionized water to completely remove the rudimental zinc chloride on the polymer surface, all the samples were freeze-dried (FD-1A-50, Beijing, China). 

The Zn–PAA composites were characterized by X-ray diffractometer (XRD, X’Pert Pro-MPD, Panalytical, Almelo, Netherlands) using Cu/Kα radiation with a wavelength of 0.154 nm at 40 kV and 200 Ma over the range of 10°–60°, Fourier transform infrared spectroscopy (FT-IR, Nicolet 6700, Waltham, MA, USA) with a spectral resolution of 1 cm^−1^ ranging from 4000 to 400 cm^−1^, and X-ray photoelectron spectroscopy (XPS, XSAM800, Manchester, UK) under the condition of an AlK alpha x-ray of 75 W and a 1000.0 meV step. The XPS spectrum was calibrated by C 1s at 284.8 eV. The morphology of the samples was observed with a scanning electron microscope (SEM, JEOL JSM-7500F, Tokyo, Japan) after being gold sputter-coated, and the acceleration voltage was adjusted to 5 kV. The element composition was analyzed via an energy-dispersive spectrometer (EDS, HORIBA Company, Kyoto, Japan) mounted on a SEM.

### 2.2. Ion Release

The release behavior of the Zn^2+^ ion from the Zn–PAA was measured in different types of solutions. Briefly, the Zn–PAA powders (200 mg) were soaked in 32 mL of phosphate buffer solution (PBS, pH value = 7.40), simulated body fluid (SBF, pH value = 7.40) and Dulbecco’s Modified Eagle’s Medium (DMEM, Gibco, Thermo Fisher Scientific, Waltham, MA, USA), respectively, at 37 °C in a shaker for 1, 7, 14 and 21 days. At each time point, the ionic concentrations were analyzed using an atomic absorption spectrometer (AAS, SpectrAA 220FS, Palo Alto, CA, USA) with a lamp current of 2.0 mA and a wavelength of 213.9 nm, and the solution was refreshed. The background has been deducted.

### 2.3. Biocompatibility In Vitro

#### 2.3.1. Cell Culture

The mouse bone marrow stem cells (BMSCs, D1 ORL UVA[D1], No. 326441, ATCC, Chinese Academy of Sciences, Shanghai, China) were used for the cell proliferation, cell morphology and osteoblast differentiation. The basic medium is DMEM supplemented with 10% fetal bovine serum (FBS, Gibco, Thermo Fisher Scientific, Scoresby, VIC, Australia) plus 1 vol. % penicillin-streptomycin solution (Gibco, Life Technologies, Grand Island, NY, USA). The cells were cultured under a 100% humidified atmosphere with 5% CO_2_ at 37 °C, and the medium was refreshed every 3 days. The samples were sterilized using UV-irradiation.

The Transwell^TM^ 3415 (a 24-well plate with a total volume of 3.4 mL, Coning corporation, Coning, NY, USA) was used in co-cultured BMSCs with Zn–PAA and PAA. Two chambers were separated by a semi-permeable membrane with a pore size of 3.0 μm. The BMSCs were cultured in the lower chambers with a density of 1 × 10^4^ cells/mL, while the Zn–PAA and PAA were cultured in the upper chambers with a mass-volume ratio of 5 mg/mL. The basic medium submerged both two chambers in order to transfer the ions and the released micro-molecules from the materials to the cells, simulating the dynamic procedure between the materials and organism, and avoiding the influences caused by other factors, such as the surface roughness, et al.

#### 2.3.2. BMSCs Proliferation and Morphology

The Transwell plates were cultured at 37 °C in a humidified atmosphere of 5% CO_2_ for 1, 4 and 7 days. The cell proliferation was determined by a Cell Counting Kit-8 (CCK-8, KeyGen BioTech, Nanjing, China). At the setting time, 100 μL CCK-8 were added in each well and incubated for 1 h at 37 °C. The optical density (OD) of each well at 450 nm was determined using a microplate reader (Multiskan FC, Thermo Fisher Scientific, Shanghai, China). The background had been eliminated. The relative cellular viability was calculated as follows:

(1) $$\mathrm{Relative}\;\mathrm{Cellular}\;\mathrm{Viability}=\frac{OD\;Value_{sample}}{OD\;Value_{control}}\times 100\%$$

The cells cultured with the basic medium were used as the blank control, and the cellular viability value of the control group was set as 100%.

In order to observe the cell morphology, after 4 days of cultivation the cells were fixed with 4% paraformaldehyde for 20 min. Prior to the fluorescence inverted microscope (Nikon Eclipse Ti-U, Nikon Instruments (Shanghai) Co., Ltd. Shanghai, China) analysis, the fixed cells were stained with Acti-stain^TM^ 488 phalloidin (Cytoskeleton Inc., Denver, CO, USA) for 40 min and 4′, 6–diamidino–2–phenylindole (DAPI, Solarbio Beijing Co., Beijing, China) for 1 min, sequentially.

### 2.4. Osteogensis of BMSCs

The basic inducing medium was configurated with the basic medium being supplemented with 50 μmol/L l–ascorbic acid (AsAP, Sigma, St. Louis, MO, USA), 10 mmol/L *β*–sodium glycerophosphate (Sigma, St. Louis, MO, USA) and 0.1 μmol/L dexamethasone (Sigma, St. Louis, MO, USA). The Zn–PAA composites were co-cultured with BMSCs via the Transwell method, as mentioned before. The BMSC suspensions were seeded at a density of 5 × 10^4^ cells/mL in 24-well plate. After 24 h of adherence, the culture medium was replaced with the inducing mediums for up to 21 days. The medium was refreshed every 4 days, and the cells were harvested at 7, 14 and 21 days.

At each time point, the cells were rinsed three times with phosphate buffered saline (PBS; Gibco, Thermo Fisher Scientific, Waltham, MA, USA), and 1 wt. % Triton X-100 buffer (Invitrogen, Carlsbad, CA, USA) was added into each well for cell lysis. In order to lyse more completely, the cells were frozen at –80 °C for 1 h and thawed to room temperature for a three-time-repeat. The ALP activity of the BMSCs was measured using the Rat ALP Elisa Kit (Mlbio, Shanghai, China), following the manufacturer’s guidelines. The ALP activities of every composite were defined as the ALP content (mU/L) normalized to the total protein content in each well (mg/L). The total protein content of the BMSCs was measured using a Bicinchoninic Acid (BCA) Protein Assay Kit (Solarbio^TM^ Beijing Co., China), following the manufacturer’s guidelines.

The calcium nodule of the cells was stained with 1 wt. % Alizarin Red S (OriCell^TM^, Cyagen Biosciences Co., Ltd., Guangzhou, China) after 21 days of inducing cultivation. The calcium nodules mineralized in the ECM were observed through an optical microscope (Nikon Eclipse Ti-U, Nikon Instruments (Shanghai) Co., Ltd., Shanghai, China).

### 2.5. Osteoclast Differentiation of RAW264.7 

The RAW264.7 cell lines (purchased from ATCC, Chinese Academy of Sciences, Shanghai, China) were seeded with 6.25 × 10^4^ cells/well and cultured in a basic media through Transwell plates. The cells could attach and grow for 12 h before being stimulated with 25 ng/mL RANKL (Peprotech, Rocky Hill, CT, USA) [25]. The experiment was carried out for 5 days. The medium was refreshed every day. Three independent trials were performed.

The media was removed and cells were washed with PBS three times. The cells were fixed with 4% paraformaldehyde. Then, they were stained with the TRAP kit (Solarbio^TM^ Beijing Co., Beijing, China), according to the manufacturer’s protocol. Positive TRAP cells were visualized and counted using Nikon Eclipse Ti-U. An osteoclast was quantified under light microscopy if it had three or more nuclei. Five different areas of a well were taken into consideration, counting the number of multicore macrophages. The relative osteoclasts’ formation rate was calculated as:

(2) $$\mathrm{Relative}\;\mathrm{Osteoclasts}\;\mathrm{formation}\;\mathrm{rate}=\frac{Number\;of{\;\mathrm{osteoclast}}_{sample}}{Number\;of{\;\mathrm{osteoclast}}_{control}}\times 100\%$$

### 2.6. Antibacterial Activity

To investigate the antibacterial activity of the simonkolleite coating, Zn–PAA and PAA were analyzed for their antibacterial activity by the growth inhibition method using Gram-negative *Escherichia coli* (*E. coli*, CICC23689) and Gram-positive *Staphylococcus aureus* (*S. aureus*, CICC21648). A sterile Tryptic Soy Broth (TSB) broth culture-medium (10 mL) and EC broth culture-medium (10 mL) were used for the activation of *S. aureus* and *E. coli*, respectively. Then, the bacterial suspensions were incubated overnight at 37 °C. A phosphate buffer contained 0.1% twain-80 (PBSt) was prepared.

The implants of different groups (0.5 g each) were sterilized by UV radiation and immersed with 95 mL PBSt and 0.5 mL E. coli and S. aureus bacterial suspensions, respectively, in a conical flask and then incubated for 24 h at 37 °C. Then, a 1 mL aliquot of each sample was plated on 3M^TM^ Petrifilm^TM^ 6406 (3M Microbiology Products, St Paul, MI, USA) and incubated at 37 °C for 24 h, following the manufacturer’s instruction. The colony forming unit (CFU) was identified and recorded by calculating the pink spots revealed on the plates. A total of three parallel experimental groups were set up. The percentage inhibition of the bacterial growth was calculated as:

(3) $$\%\mathrm{Growth}\;\mathrm{inhibition}=\;\frac{CFUs\;of\;control-\;CFUs\;of\;text\;sample}{\;CFUs\;of\;control}\;\times 100$$

### 2.7. Statistical analysis

The results of the relative cellular viability, ALP activity, relative osteoclasts’ formation rate and the antibacterial growth inhibition were performed with a one-tailed Student’s t-test. The EDS results were presented as the mean ± standard deviation. The statistical significance was set at a value of p < 0.05.

## 3. Results

### 3.1. Characterization of PAA and Zn-PAA

**Figure 1:**
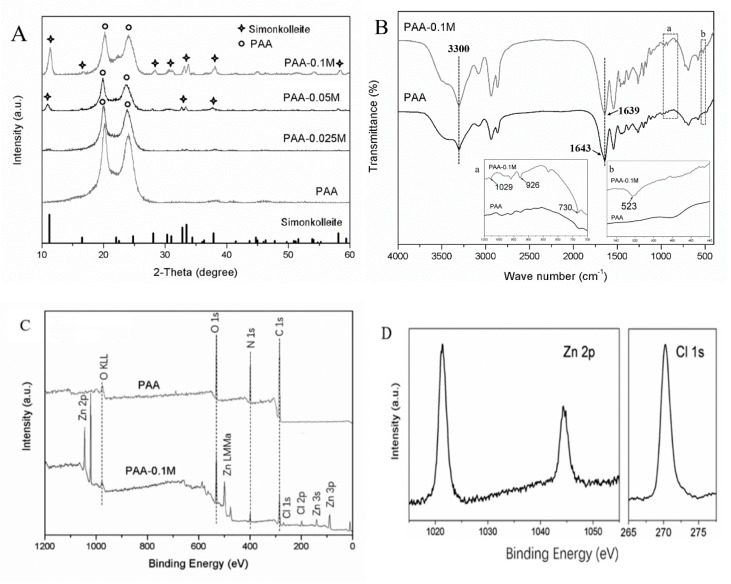
(**A**) The XRD patterns of PAA–0.1M, PAA–0.05M, PAA–0.025M and PAA (the simonkolleite spectrum was derived from the standard PDF card No. 00-07-0155); (**B**) The FT-IR patterns of PAA–0.1M and PAA: (a) the enlarged view from wavenumbers 1050 to 700 cm^−1^, (b) the enlarged view from wavenumbers 550 to 440 cm^−1^; (**C**) The XPS spectrum of PAA–0.1M and PAA; and (**D**) the enlarged Zn 2p and Cl 1s regions of PAA–0.1M.

The XRD spectrums of Zn–PAA are shown in Figure 1A. The peaks at 20.0° and 23.6° demonstrated the α-phase of polyamide 6 incorporating in hypo-crystalline poly (amino acids) [26]. The other peaks at 10.9°, 27.9°, 31.6°, 32.7°, 33.4°, 37.6° and 56.2° contributed to the typical hexagonal nanosheets of simonkolleite, Zn_5_(OH)_8_Cl_2_·H_2_O (PDF 00-07-0155) [27]. No signs of zinc chloride, zinc hydroxide or other zinc salts appeared, indicating that only simonkolleite deposed on the surface of the PAA. Moreover, the intensity of the characteristic peaks of simonkolleite, which corresponded to the content of simonkolleite on the surface of the composites, gradually diminished from PAA–0.1M to PAA–0.025M. The content of the simonkolleite corresponded to the concentration of the ZnCl_2_ immersing solutions, the only variation among the three Zn–PAA during the preparation process. When the ZnCl_2_ concentration was below 0.025 mol/L (PAA–0.025M), the simonkolleite content was too low to be identified from the XRD.

The FT-IR spectrums of PAA and PAA–0.1M (Figure 1B) had approximately similar patterns that have strong bands at about 3300 cm^−1^, corresponding to the stretching vibration of N–H in the PAA phase. In PAA–0.1M, the weak bands at 1029, 926, 730 and 523 cm^−1^ illustrated the presence of simonkolleite [28,29]. The characteristic peaks corresponding to the C=O stretching vibration band (amide I) was at 1643 cm^−1^ in PAA, and it was blue shifted to 1639 cm^−1^ in PAA–0.1M, suggesting the generation of a hydrogen bond between the PAA phase and the simonkolleite phase. 

The XPS spectrum (Figure 1C,D) of PAA–0.1M and PAA exhibited the existence of zinc and chloride in PAA–0.1M. The Zn 2p spectrum showed a doublet with an expected branching ratio of 2:1, which was a typical sign of simonkolleite [18]. The Zn 2p3/2 and Zn 2p1/2 had a binding energy of 1021.3 eV and 1044.3 eV, which could be assigned to Zn–OH and Zn–Cl, respectively. The peak of the Cl 1s spectrum at 271.2 eV further indicated the presence of Zn–Cl. These peaks also supported the existence of simonkolleite. A slight shift of the O KLL spectrum from 974.5 eV in PAA to 978.5 eV in PAA–0.1M was observed, which might indicate the generation of hydrogen bonds, coherent with the FT-IR results.

### 3.2. Morphology and Composition of PAA and Zn–PAA

**Figure 2:**
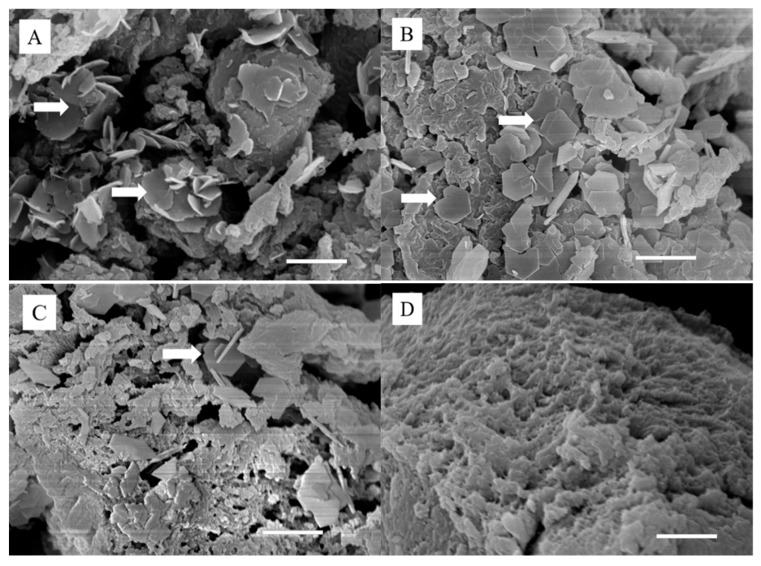
The SEM analysis of the surface morphology of (**A**) PAA–0.1M, (**B**) PAA–0.05M, (**C**) PAA–0.025M and (**D**) PAA (white arrows represent simonkolleite crystals, the scale bar: 2 μm).

The SEM micrographs of Zn–PAA (Figure 2) exhibited deposits with a hexagonal and platelet-like morphology, which was similar to that of simonkolleite, as reported by other researchers [16,30,31], while the surface of PAA had no deposits. Meanwhile, the ZnCl_2_ concentration of the immersed solutions was positively correlated with the amount of simonkolleite deposited on the PAA, correlating with the XRD results.

The compositions of Zn–PAA characterized by EDS are shown in Table 1. The atomic ratio of Zn to Cl was 2.85 in PAA–0.1M, 2.75 in PAA–0.05M and 2.13 in PAA–0.025M, which was close to that in simonkolleite. Furthermore, the zinc content dropped from 0.97 at % in PAA–0.1M to 0.17 at % in PAA–0.025M, revealing a decrease of the simonkolleite content associated with the decreased concentration of zinc chloride solution used in the preparation process, in line with the XRD results.

### 3.3. Zinc Ion Release in Different Solutions

**Figure 3:**
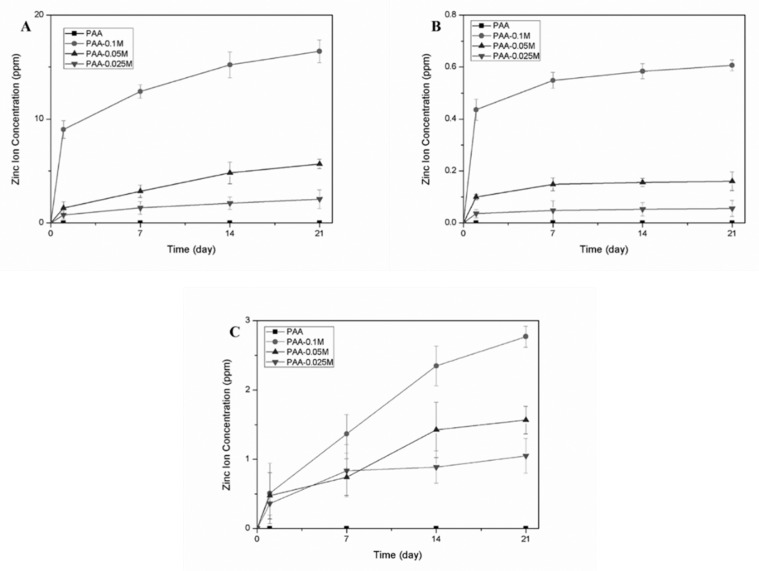
The zinc ion release of PAA, PAA–0.1M, PAA–0.05M and PAA–0.025M in (**A**) DMEM, (**B**) PBS and (**C**) SBF after 1, 7, 14 and 21 days, as detected by AAS.

The release of Zn^2+^ from the composites after 1, 7, 14 and 21 days of immersion in PBS, SBF and DMEM was measured by AAS. As shown in Figure 3, the released Zn^2+^ increased gradually within the immersion time and was positively correlated with the simonkolleite content in all types of solutions. In PBS and DMEM, the Zn^2+^ release was rather acute for 1 day and became mild during up to 21 days, compared to which the zinc exhibited a sustained release behavior during 14 days in SBF, and the initial burst release was extremely suppressed. It was worth noting that the accumulated concentration of released Zn^2+^ differed among the solutions: the maximal Zn^2+^ concentration of PAA–0.1M released after 21 days in DMEM (16.5 mg/L) was much higher than that in PBS (0.6 mg/L) and in SBF (2.8 mg/L).

### 3.4. Cytocompatibility of Zn–PAA

**Figure 4:**
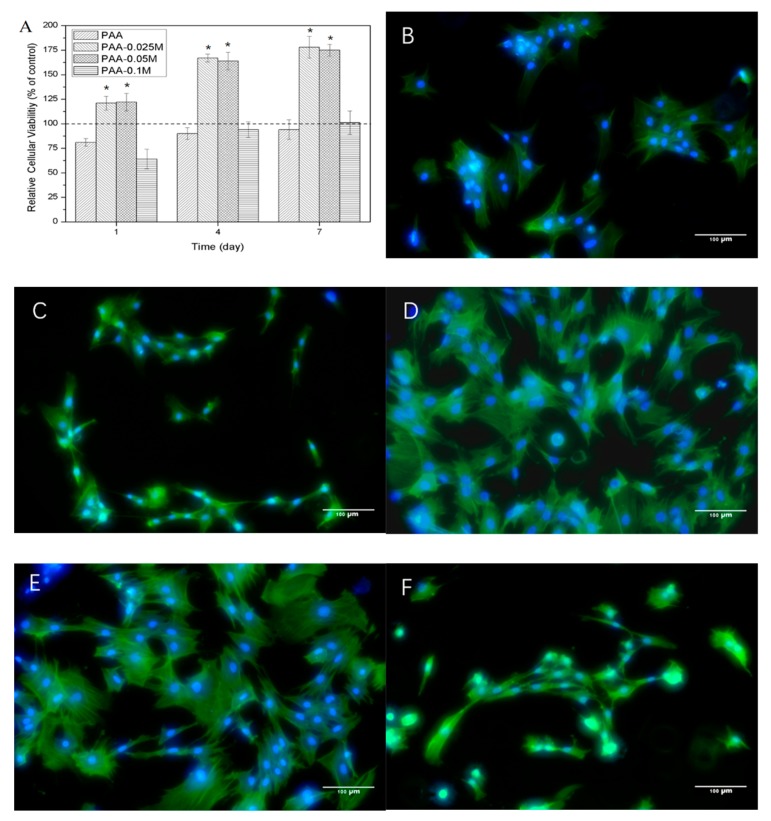
The relative cell viability and morphology of BMSCs cultured with Zn–PAA and PAA. (**A**) The cell viability of BMSCs cultured with Zn–PAA and PAA at 1, 4 and 7 days detected by cck-8 kit. (* *p* < 0.05 compared to PAA and PAA–0.1M); the morphology of BMSCs at 4 days of incubation cultured with (**B**) black control; (**C**) PAA; (**D**) PAA–0.025M; (**E**) PAA–0.05M; and (**F**) PAA–0.1M. The scale bar: 100 μm.

The proliferation of BMSCs assessed by CCK-8 are presented in Figure 4A. The cell viability for the PAA group was slightly lower than the control for all the time points, and it increased from 81% at 1 day to 94% at 7 days. When a small amount of simonkolleite was coated on the PAA (PAA–0.025M), the cell viability dramatically increased to 125% at 1 day and 178% at 7 days, and the cell viability for the PAA–0.05M group was similar. However, when the amount of simonkolleite coated on the PAA further increased (PAA–0.1M), the cell viability dropped to 64% at 1 day, followed by it soaring to 101% after 7 days. The results revealed that the response of BMSCs to the simonkolleite coating was highly dose-dependent: an appropriate dose (e.g., PAA–0.025M and PAA–0.05M) was able to promote cell proliferation, while a high dose (e.g., PAA–0.1M) exhibited cytotoxicity. 

The cell morphology of BMSCs co-cultured with Zn–PAA after 4 days is shown in Figure 4B–F. The cells with PAA and PAA–0.1M did not spread very well and exhibited a fibroblast-like colony with a narrow structure, which was in accordance with the results of the cell proliferation. The cells with PAA–0.025M and PAA–0.05M exhibited a flattened expanded shape with a clear framework and tentacles, which was similar to the control, indicating a good cytocompatibility. In addition, more cells were observed with PAA–0.025M and PAA–0.05M compared to PAA, PAA–0.1M and the control, which corresponded to the results of the cell proliferation.

### 3.5. Osteogenic Differentiation of BMSCs

**Figure 5:**
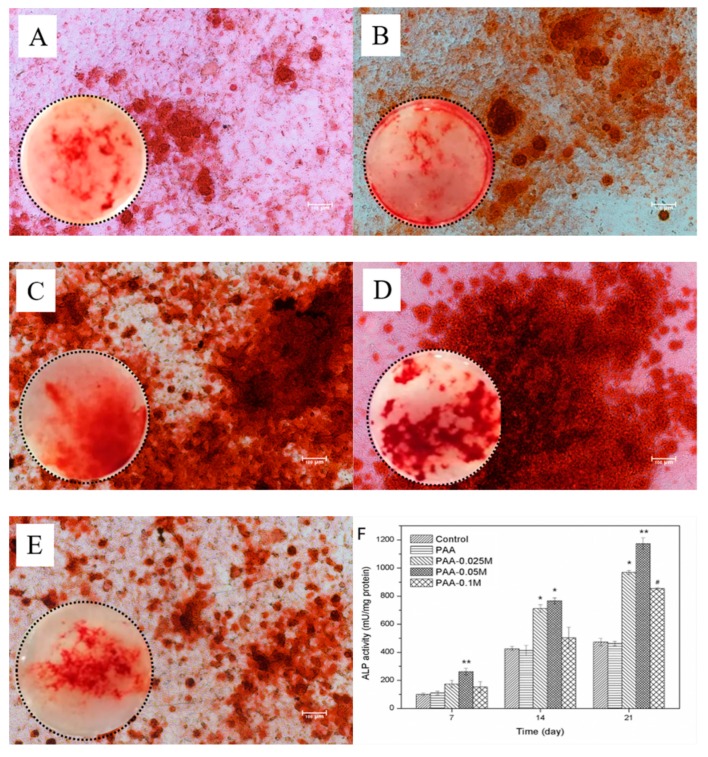
The Alizarin Red S staining of BMSCs at 21 days under an optical microscope, and the ALP activity of BMSCs cultured with Zn–PAA and PAA for 7, 14 and 21 days. The Alizarin Red S staining of the BMSCs cultured with (**A**) control, (**B**) PAA, (**C**) PAA–0.025M, (**D**) PAA–0.05M and (**E**) PAA–0.1M; the scale bar: 100 μm; and their photographs in the Transwell plate under chambers (inserted); (**F**) the ALP activity of BMSCs (** *p* < 0.05 compared to each other sample; * *p* < 0.05 compared to the control, PAA or PAA–0.1M; # *p* < 0.05 compared to the control or PAA).

The ALP activity of BMSCs co-cultured with Zn–PAA and PAA for 21 days was assessed, and the result is shown in Figure 5F. The PAA–0.025M and PAA–0.05M exhibited a higher ALP activity compared to PAA and PAA–0.1M. The highest ALP activity was found for PAA–0.025M for all the time points. In addition, the PAA group showed a rapidly increased ALP activity in the initial 14 days and then tended to be steady, while the ALP activity of the Zn–PAA groups kept increasing during the cultivation time. These results indicate that the appropriate concentration of continuously released zinc intrinsically improved and supported the osteogenic differentiation of BMSCs. The mineralization of BMSCs cultured with PAA and Zn–PAA after 21 days was evaluated by Alizarin red staining (Figure 5A–E). More red-stained calcium nodules were found in all Zn–PAA groups than in PAA group, indicating that simonkolleite coating could promote the mineralization of BMSCs. The largest quantity of calcium nodules was found in the PAA–0.025M and PAA–0.05M groups instead of the PAA–0.1M group, which was consistent with the results of the ALP activity, suggesting that the effect of simonkolleite coating on cell mineralization was also dose-dependent and that a suitable dose was crucial.

### 3.6. Osteoclast Differentiation of RAW264.7

**Figure 6:**
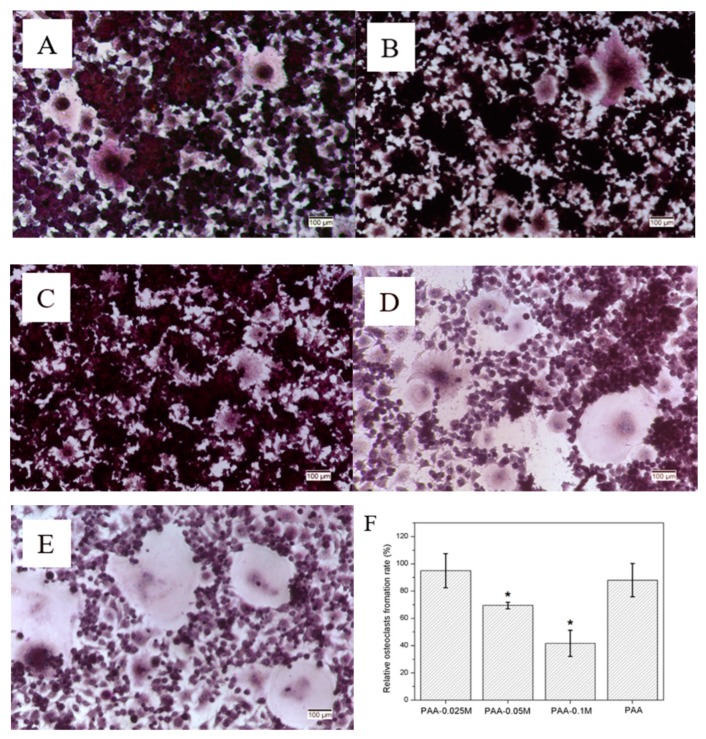
RAW264.7 cultured with (**A**) PAA–0.025M; (**B**) PAA–0.05M; (**C**) PAA–0.1M; (**D**) PAA and (**E**) control in a Transwell plate stimulated by 25 ng/mL RANKL at 5 days under an optical microscope; the scale bar: 100 μm; and (**E**) the relative osteoclasts’ formation rate of the Zn–PAA and PAA groups. (* *p* < 0.05).

To investigate the effect of simonkolleite coating on osteoclastogenesis, which counteracted with osteogenesis and is undesirable in bone regeneration [32], a RAW264.7 cell line was used and induced to differentiate into osteoclasts by the addition of RANKL in the presence of PAA or Zn–PAA. Photographs of representative cells are presented in Figure 6A–D. Osteoclasts with enormous sizes and multiple nuclei were found in the PAA and control groups, and they were smaller and fewer with the increase of the simonkolleite content in the Zn–PAA groups. Moreover, osteoclasts that differentiated from RAW264.7 were quantitated after staining for TRAP, a specific marker of the osteoclast phenotype, and the relative osteoclasts’ formation rate is presented in Figure 6E. The osteoclast formation was significantly and dose-dependently suppressed by the simonkolleite content in Zn–PAA, while the effect of PAA was not observed. These results suggested that the simonkolleite coating on the PAA was able to block the osteoclast differentiation of RAW264.7 cells in a dose-dependent manner, thus contributing to supporting osteogenesis.

### 3.7. Antibacterial Activity

**Figure 7:**
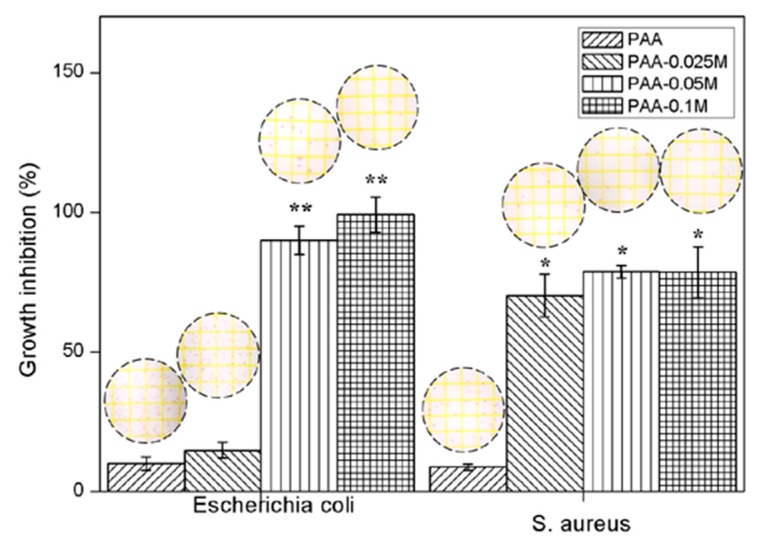
The inhibition rate of Zn–PAA and PAA against *E. coli* and *S. aureus* after 24 h of cultivation. (* *p* < 0.05 compared to PAA, ** *p* < 0.05 compared to PAA–0.025M and PAA).

The inhibition rate of *E. coli* and *S. aureus* in the presence of the samples after 24 h incubation is displayed in Figure 7. The inhibitory rates of PAA against both *E. coli* and *S. aureus* were extremely low (about 10% and 8%, respectively), indicating almost no antimicrobial activity. PAA–0.025M exhibited a high antibacterial activity against *S. aureus* but almost no antimicrobial activity against *E. coli*. Both PAA–0.05M and PAA–0.1M showed a high bacterial inhibition against *E. coli* and *S. aureus*, while no significant difference was found between them. The results demonstrated that the simonkolleite coating could remarkably improve the antibacterial activity of Zn–PAA against *E. coli* and *S. aureus*, when the content of simonkolleite was adequate.

## 4. Discussion

Zinc has been reported to be crucial in regulating the homeostasis of bone by stimulating osteoblasts’ proliferation and differentiation to improve bone formation as well as by inhibiting osteoclasts’ activity to avoid bone resorption [33]. Osteoblasts cultured with an appropriate amount of zinc showed a significantly increased proliferation, which might be related to protein syntheses [34], along with the raising of the alkaline phosphatase activity [35]. Thus, bone regeneration materials, which serve as a zinc resource and can continuously release zinc in an appropriate manner, are particularly beneficial in enhancing the bone-repairing performance.

We used simonkolleite, which was coated on PAA by immersing PAA powders into a ZnCl_2_ solution, as a novel zinc resource for osteogenesis. The simonkolleite coatings on the polymer were observed by SEM (Figure 2) as hexagonal structure crystals staged together and were characterized by XRD (Figure 1A) as characteristic peaks at 10.9°, 27.9°, 31.6°, 32.7°, 33.4°, 37.6° and 56.2° [36], along with the XPS (Figure 1D) of the typical binding energy of Zn 2p3/2 and Zn 2p1/2 at 1021.3 eV and 1044.3 eV, respectively. Moreover, the shifted C=O stretching vibration band in the FT-IR spectrum (Figure 1B) and the slightly shifted O KLL component in XPS suggested that hydrogen bonds were generated between the coating and PAA phase [37,38]. More significantly, the total amount of simonkolleite coating was associated with the concentration of the ZnCl_2_ immersing solution, as proven by the SEM and EDS tests (Table 1), which could be used to regulate the content of the coating.

The coated Zn^2+^ ions must depart from the composites and enter the fluid environment to be able to interact with the cells. Three different mediums (DMEM, PBS and SBF) were used to evaluate the zinc ion release of the synthesized composites with different simonkolleite contents. The results revealed that the quantities of zinc released were associated with both the total amount of simonkolleite coating and the type of soaking medium. Zn–PAA rarely released Zn^2+^ ions in PBS, which might be attributed to the insolubility of simonkolleite in water. Interestingly, the total ion concentration varied between the three type of solutions, so the zinc release of simonkolleite might be associated with the ions in the medium, either by reactions or by ion-exchange.

The reason why we put so much effort into the zinc release is that the concentration of zinc ions is crucial for osteoblast proliferation, differentiation and mineralization. Zaveri et al. [39] discovered that the cytotoxicity of bone marrow-derived macrophages appeared when the concentration of released Zn^2+^ ions was over 150 μM. Song et al. [40] reported that the cytotoxicity of Ana-1 cells appeared when the zinc concentration was around 5–10 μg/mL. Ito et al. [41] discovered that the maximal zinc concentration of non-cytotoxicity of MC3T3-E1 was around 3 ppm. In our research, when the zinc concentration in the culturing media rose (derived from an increased simonkolleite coating content in Zn–PAA), the relative cellular viability (Figure 4A), the ALP activity (Figure 5F) and the amount of calcium nodules (Figure 5A–E) responded in a dose-dependent way. The highest relative cellular viability, ALP activity and calcium nodules were found with PAA–0.05M (the zinc concentration ranged from 1.44 to 5.16 μg/mL), as these were much higher than those with PAA and the control. Nevertheless, cytotoxicity was observed with PAA–0.1M (the zinc concentration ranged from 9.0 to 16.5 μg/mL), along with a decreased ALP activity and quantity of calcium nodules. These results demonstrated that the proliferation, differentiation and mineralization of BMSCs could be remarkably enhanced when the concentration of released Zn^2+^ was suitable (PAA–0.05M), while they could be impeded when it was too high (PAA–0.1M), suggesting that the zinc release behavior was crucial for Zn–PAA to promote osteogenesis.

Bone homeostasis is mainly regulated by the balance between osteoclastogenesis and osteoblastogenesis [42,43]. Zinc transporters are expressed in osteoclasts, and some are up-regulated during osteoclast differentiation, suggesting that zinc plays an important role in osteoclast differentiation [44]. The inhibition of osteoclast formation by zinc was proven to be dose-dependent, but the initial inhibition concentration of zinc was different among studies. For instance, Yamaguchi [45] found that osteoclast formation was dose-dependently suppressed by zinc sulfate in the range of 10–250 μM by exhibiting fewer osteoclasts and no effect on the proliferation of RAW264.7; Luo [4] discovered that the zinc released from zinc-containing TCP could induce both the proliferation and differentiation of RAW264.7 cells into osteoclasts when the zinc concentration was below 18 ppm; Yu [46] illustrated that the proliferation of RAW264.7 cells was suppressed by released zinc from Zn-modified calcium silicate ceramic and that no fusion of mononuclear cells into osteoclasts was observed. In our study, RAW264.7 cells, being easy to culture and sensitive to RANKL stimulation, were used to evaluate the osteoclastogenesis of Zn–PAA through Transwell [47,48]. The results confirmed that the osteoclast formation rate decreased as the content of simonkolleite rose, which could be attributed to the released Zn^2+^ ion in the medium. When the simonkolleite content is extremely high (PAA–0.1M), resulting in a high Zn^2+^ concentration, almost no osteoclast formation was observed. The inhibiting effect of PAA–0.05M on the osteoclast formation was also prominent, while that of PAA–0.025M was not observed, indicating that an adequate simonkolleite content was necessary to suppress osteoclastogenesis.

In the initial bone repair period, inflammation occurred, forming hematoma and an influx of fibrous granulation tissues around defects, which is detrimental to osteogenesis [49]. Simonkolleite, as a zinc supplementation, may be a simple, cheap and effective anti-inflammatory agent with multiple pathological conditions by repressing the activation of NF-κB, an important signal transduction in the initiation and maintenance of inflammatory responses [45]. 

In recent years, the interest in functionalizing orthopedic implants with antimicrobial activity to prevent postoperative infection has grown. Previous studies have proven the excellent antibacterial activity of soluble zinc compounds against various bacterial and fungal strains [50]. The antimicrobial mechanism of zinc ions is not yet fully understood, but it is mainly associated with two effects. One is that zinc can break the membrane stabilization of microorganisms, leading to an increased permeability by directly interacting with microbial membranes [51], and the other is attributed to its interaction with the microbial nucleic acids and deactivation enzymes of the respiratory system [52]. In our research, the antibacterial activity of Zn–PAA against *E. coli* and *S. aureus* was investigated, and the results revealed that the release of Zn^2+^ ions was mainly responsible for the enhanced antimicrobial effect of Zn–PAA, highly dependent on bacterial strains and the dose of simonkolleite. Zn–PAA with an adequate simonkolleite content (PAA–0.05M and PAA–0.1M) showed an excellent antibacterial effect against both two bacteria, while an insufficient simonkolleite content could result in an inapparent antibacterial effect (PAA–0.025M).

While taking comprehensive consideration of its capacity to stimulate bone formation, repress bone resorption, and antibacterial activity, Zn–PAA with an appropriate simonkolleite content (e.g., PAA–0.05M) is a promising multifunctional bone repair material and shows a great potential for application.

## 5. Conclusions

Simonkolleite coating was fabricated on PAA-forming Zn–PAA to promote osteogenesis by immersing PAA in a ZnCl_2_ solution, and the amount of coating could be adjusted by regulating the ZnCl_2_ concentration of the immersing solution. Zn–PAA could continuously release Zn^2+^ ions according to both the simonkolleite content and the type of soaking solution. By introducing simonkolleite coating to PAA, its ability to promote the proliferation, differentiation and mineralization of BMSCs could be enhanced in a dose-dependent manner. The maximal proliferation, differentiation and mineralization were found with PAA–0.05M, while PAA–0.1M exhibited a mild cytotoxicity, hindered differentiation and reduced mineralization. The simonkolleite coating also enhanced the performance of Zn–PAA in inhibiting osteoclastogenesis in vitro, which was favorable for the early stages of bone regeneration. Additionally, Zn–PAA with an adequate simonkolleite content (PAA–0.05M and PAA–0.1M) had an excellent antibacterial activity against both *E. coli* and *S. aureus*, which is important in preventing a post-operative infection. These results offered a novel way to functionalize synthetic bone graft material and to identify the underlying principles for designing zinc-containing bone grafts.

## Figures and Tables

**Table 1:** The EDS data of the PAA–0.1M, PAA–0.05M and PAA–0.025M surfaces.

Samples/Elements	C (at %)	O (at %)	Zn (at %)	Cl (Atomic %)
PAA–0.1M	79.76 ± 0.42	18.94 ± 0.03	0.97 ± 0.01	0.34 ± 0.03
PAA–0.05M	79.69 ± 0.31	19.84 ± 0.06	0.33 ± 0.01	0.12 ± 0.02
PAA–0.025M	78.12 ± 0.19	20.91 ± 0.14	0.17 ± 0.01	0.08 ± 0.02
